# IKKα regulates the stratification and differentiation of the epidermis: implications for skin cancer development

**DOI:** 10.18632/oncotarget.12527

**Published:** 2016-10-08

**Authors:** Josefa P. Alameda, Manuel Navarro, Ángel Ramírez, Angustias Page, Cristian Suárez-Cabrera, Rodolfo Moreno-Maldonado, Jesús M. Paramio, María del Carmen Fariña, Marcela Del Río, María Jesús Fernández-Aceñero, Ana Bravo, María de los Llanos Casanova

**Affiliations:** ^1^ Molecular Oncology Unit, Centro de Investigaciones Energéticas, Medioambientales y Tecnológicas (CIEMAT), Madrid, Spain; ^2^ Molecular Oncology, Institute of Biomedical Investigation University Hospital “12 de Octubre”, Madrid, Spain; ^3^ Present address: SILO España, Madrid, Spain; ^4^ Department of Dermatology, Fundación Jiménez Díaz, Madrid, Spain; ^5^ Epithelial Biomedicine Division, CIEMAT-CIBERER (U714), Madrid, Spain; ^6^ Department of Bioengineering, Carlos III University (UC3M), Leganés, Madrid, Spain; ^7^ Cátedra Fundación Jiménez Díaz (IIS-FJD) of Regenerative Medicine and Tissue Bioengineer, Madrid, Spain; ^8^ Department of Pathology, Hospital Clínico San Carlos, Madrid, Spain; ^9^ Department of Veterinary Clinical Sciences, Faculty of Veterinary Medicine, University of Santiago de Compostela, Lugo, Spain

**Keywords:** IKKα, keratinocyte differentiation, MMP9, skin, skin cancer

## Abstract

IKKα plays a mandatory role in keratinocyte differentiation and exerts an important task in non-melanoma skin cancer development. However, it is not fully understood how IKKα exerts these functions. To analyze in detail the role of IKKα in epidermal stratification and differentiation, we have generated tridimensional (3D) cultures of human HaCaT keratinocytes and fibroblasts in fibrin gels, obtaining human skin equivalents that comprise an epidermal and a dermal compartments that resembles both the structure and differentiation of normal human skin. We have found that IKKα expression must be strictly regulated in epidermis, as alterations in its levels lead to histological defects and promote the development of malignant features. Specifically, we have found that the augmented expression of IKKα results in increased proliferation and clonogenicity of human keratinocytes, and leads to an accelerated and altered differentiation, augmented ability of invasive growth, induction of the expression of oncogenic proteins (Podoplanin, Snail, Cyclin D1) and increased extracellular matrix proteolytic activity. All these characteristics make keratinocytes overexpressing IKKα to be at a higher risk of developing skin cancer. Comparison of genetic profile obtained by analysis of microarrays of RNA of skin equivalents from both genotypes supports the above described findings.

## INTRODUCTION

The epidermis is a stratified squamous epithelium composed mainly of keratinocytes. Basal keratinocytes proliferate and give rise to differentiated cells, which, upon full maturation, generate the squamous cornified cell layer. Alterations in the normal proliferation/differentiation equilibrium of the epidermis lead to numerous pathologies, which is important because skin disorders are the most frequent pathologies in humans. Different studies performed in knockout mice for IKKα or in mice with epidermal keratinocyte-specific IKKα ablation have concluded that IKKα is essential for epidermal differentiation [1–4]. IKKα is a member of the IKK complex, which is composed of two kinase subunits, IKKα and IKKβ, and a regulatory subunit, IKKγ/NEMO. Activated IKK complexes phosphorylate IkBα, leading to its ubiquitination and degradation and to the subsequent activation of NF-κB [5, 6]. It has been described that the function of IKKα in epidermis is independent of its kinase activity regulating NF-κB [7], although the early death of IKKα null mice after birth precludes the study of many aspects related to mechanisms through which IKKα controls epidermal differentiation. In this regard, it has been recently proposed that the induction of IKKα has an important role in the pathogenesis of skin diseases that course with altered proliferation/differentiation equilibrium, such as psoriasis [8], suggesting an important role of IKKα in the maintenenace of the homeostasis of the epidermis in humans.

IKKα has also been connected with non-melanoma skin cancer (NMSC), although there are studies that suggest that IKKα may act as a tumor suppressor [9, 10] or as a tumor promoter [11, 12] in this type of cancer, and this controversy has not yet been solved. Therefore, the development of a new experimental model to decipher the mechanisms through which IKKα regulates the homeostasis of the epidermis and the development of non-melanoma skin cancer is necessary.

In the present study we have employed a new approach to solve these questions, using a new model of study, based on the generation of 3D co-cultures of HaCaT keratinocytes and skin fibroblasts embedded in fibrin gels. These cultures eventually give rise to an ordered structure equivalent to human skin (to which we refer to as skin equivalent), that comprise an epidermal and a dermal compartment that mimic a normal human skin [13]. The spontaneously immortalized human HaCaT cell line has been widely used in studies related to keratinocytes and epidermal biology because it maintains full epidermal differentiation capacity [13, 14]. Our previous studies showed that enhanced IKKα expression in HaCaT cells increased both early and terminal differentiation of these cells in differentiation assays in monolayer cultures [12]. However, in these kind of cultures the formation of basal and suprabasal layers is not possible; therefore, they do not allow for the sequential study of the epidermal stratification process. Our present approach using skin equivalents of HaCaT keratinocytes allows the study, in more physiological conditions, of the contribution of IKKα to the differentiation of the distinct epidermal layers. As a result, we have found that increased levels of IKKα accelerates the differentiation of human keratinocytes, although in an aberrant form, leading to the development of histological defects. In addition, our analysis shows that the augmented expression of IKKα promotes the appearance of preneoplastic changes and the expression of oncogenic proteins in human keratinocytes. The comparison of the genetic profiles obtained by analysis of microarrays of RNA of skin equivalents of both genotypes support these findings. In addition they show the up-regulation in the HaCaT-IKKα skin equivalents of genes also up-regulated in different skin diseases, such as psoriasis and ichthyosis, and in skin cancer. These results highlight the usefulness of our *in vitro* model of skin equivalents for studying the physiology and disorders of the skin.

## RESULTS

### Increased levels of IKKα induces dysplastic changes, disorganized stratification and altered differentiation in human skin equivalents

**Figure 1:**
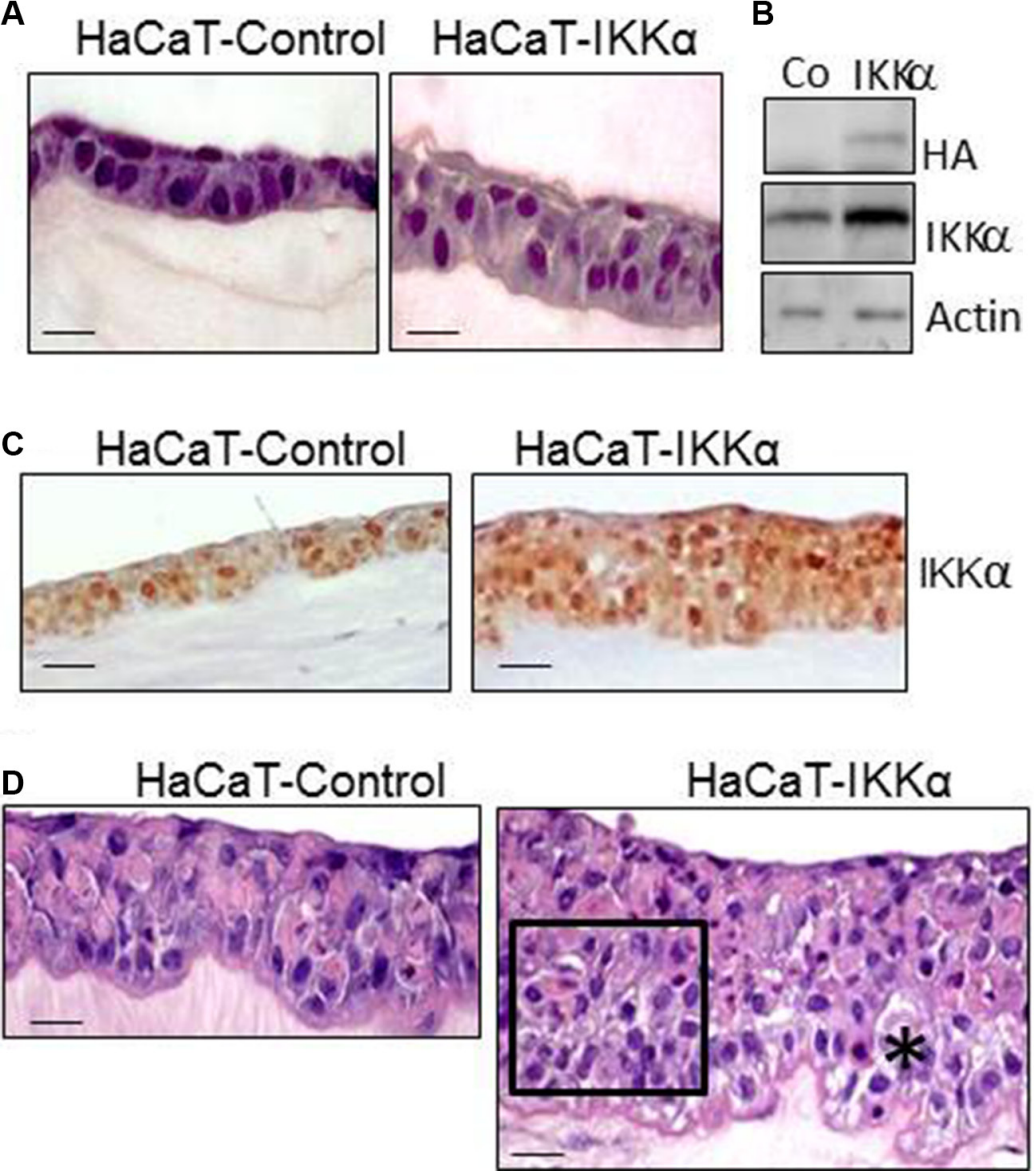
Histological characterization of HaCaT-Control and HaCaT-IKKα skin equivalents (**A**) Appearance of skin equivalents after 2 days of differentiation in air-liquid interface culture. Note the increase in the number of keratinocyte layers in the HaCaT-IKKα 3D cultures. (**B**) Western blot showing the expression of the exogenous IKKα in the HaCaT-IKKα skin equivalents (protein extracts derived from 2-day fibrin gels). Actin was used as a loading control. (**C**) Immunostaining showing IKKα expression in the HaCaT-Control and HaCaT-IKKα skin equivalents using the NB-100-56704 antibody. (**D**) 12-day skin equivalents showing the increased stratification and marked morphological alterations found in the HaCaT-IKKα cultures. (*) = area of spongiosis; rectangle = area of disorganized keratinocytes. Scale bar: 30 μm (A, D); 50 μm (C).

We have used the HaCaT-Control and HaCaT-IKKα cell populations of keratinocytes previously described [12] to generate skin equivalents. HaCaT-IKKα cells express the mouse IKKα cDNA under the control of the β-actin promoter and HaCaT-Control cells contain the empty vector. Both HaCaT-Control and HaCaT-IKKα keratinocytes were seeded on a fibrin matrix. Two to three days later they reached confluence and were raised to the air-liquid interface for up to 12 additional days to generate a stratified, differentiated epidermis (confirmed by histological and immunohistochemical analysis). Figure 1A shows the histological appearance of the fibrin organotypic skin equivalent established from HaCaT-Control keratinocytes and cultured for 2-days at the air-liquid interface. As shown, distinctive features that are normally seen in the epidermis *in vivo* can readily be distinguished, including well-organized and defined epidermal cell layers (basal and suprabasal). Histological resemblance with a human epidermis was also observed in HaCaT-IKKα skin equivalents (Figure 1A). The expression of the transgene in the HaCaT-IKKα skin equivalents was verified by western blot and immunohistochemistry (Figure 1B, 1C). The histological analysis showed that HaCaT-IKKα keratinocytes stratified faster than HaCaT-Control cells, as higher number of cell layers were observed in their epidermal compartment from 2-days of air-liquid culture onward (Figure 1A, 1D). Thus, while 2-day skin equivalents of HaCat-Control cells showed one basal and one suprabasal layer (this latter readily distinguished by the presence of keratinocytes with flattened nucleus), in 2-day HaCaT-IKKα equivalents there were 3 to 4 cell layers of keratinocytes, organized into three distinct strata: basal stratum (formed by a layer of cylindrical cells containing large nuclei), suprabasal stratum (with 1 or 2 layers of cells with smaller nuclei), and an upper stratum formed by cells with flattened nuclei (Figure 1A). HaCaT-Control skin equivalents of 6 to 12 days of differentiation showed 3 to 5 keratinocyte layers. By contrast, the HaCaT- IKKα skin equivalents exhibit up to 7–11 layers on day 12 (Figure 1D). In addition, we found that the stratification of the HaCaT- IKKα skin equivalents was disorganized, showing disorientated nuclei; they also presented dysplastic keratinocytes in large areas of the epidermis. These defects were similarly detected in the epidermis of transgenic mice expressing exogenously IKKα in the basal layer of the epidermis (K5-IKKα mice) in conditions of hyperproliferation [11]. Areas of spongiosis (intercellular edema of the epithelium) were also detected in the basal as well as in the suprabasal layers of HaCaT-IKKα skin equivalents (Figure 1D), being this alteration also detected in the epidermis of transgenic K5- IKKα mice (data not shown). By contrast, the stratification of HaCaT-Control skin equivalents resembled that of a normal human skin (Figure 1D).

**Figure 2:**
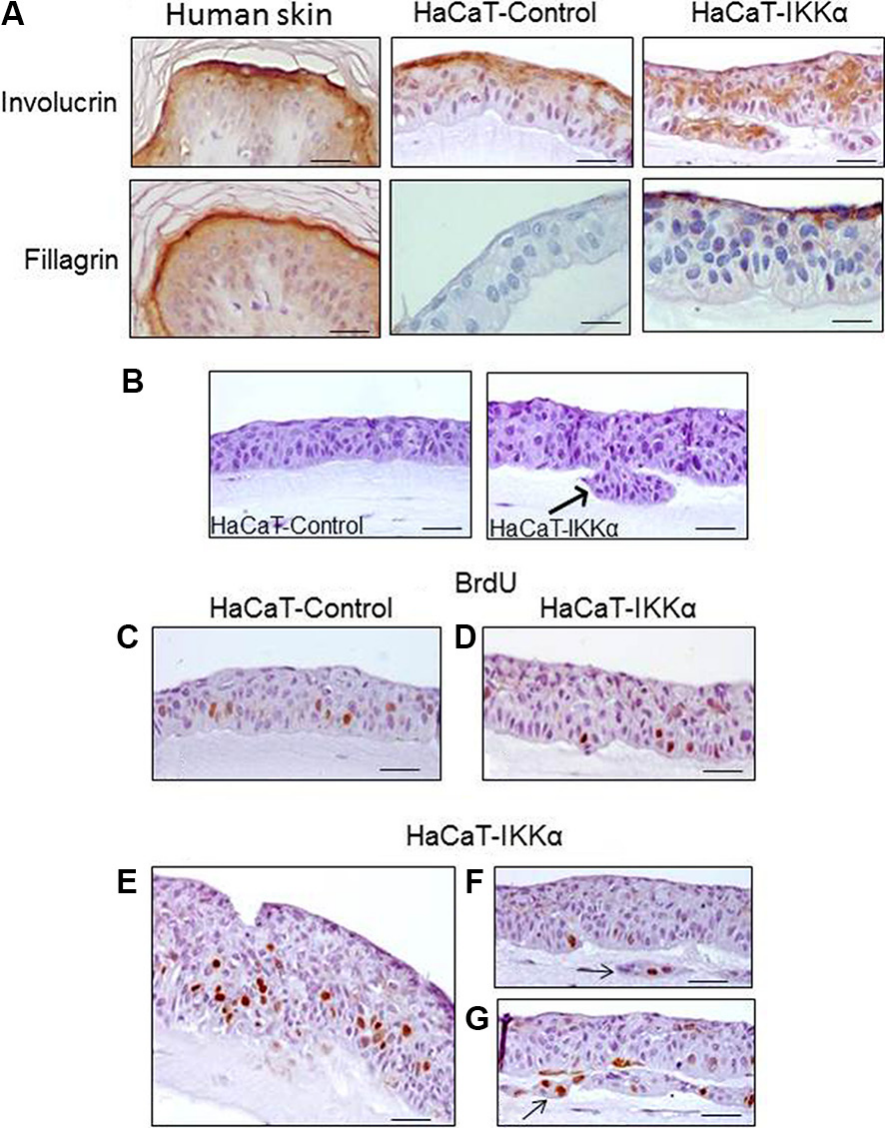
Altered differentiation, increased proliferation and invasion foci in the HaCaT-IKKα skin equivalents (**A**) Immunostaining with two markers of epidermal differentiation; involucrin (upper panel) and filaggrin (a marker of upper terminal epidermal differentiation), (lower panel). The expression of these proteins in normal human epidermis as well as in HaCaT Control and HaCaT-IKKα skin equivalents is shown. (**B**) Invasive foci are observed in the 3D cultures of HaCaT-IKKα keratinocytes (arrow). (**C**–**G**) Representative examples of BrdU incorporation. (C) HaCaT-Control skin equivalents: observe the predominance of signal in the basal layer; (D–G) BrdU signal in HaCaT-IKKα skin equivalents. (D) The BrdU staining in areas with light stratification defects in the HaCaT-IKKα skin equivalents is similar to that of HaCaT-Control (C); (E) BrdU incorporation in basal as well as suprabasal layers of keratinocytes is detected in areas where HaCaT-IKKα equivalents exhibit more aberrant stratification; (F–G) BrdU positive signal are detected in foci of invasion (arrows). Scale bar: 30 μm (A); 40 μm (C–G); 50 μm (B).

In line with the stratification defects of HaCaT-IKKα keratinocytes in the bioengineered skin, the expression of involucrin (a protein characteristic of suprabasal layers and commonly used as a marker of early epidermal differentiation) was altered in the epidermal compartment of the HaCaT-IKKα skin equivalents, being delocalized along all the keratinocyte layers, including the basal layer (Figure 2A). By contrast, it was correctly localized in the suprabasal layers of the HaCaT-Control skin equivalents, following the pattern of expression observed in the normal human epidermis (Figure 2A). We also observed that the increased expression of IKKα seems to favor the terminal differentiation of keratinocytes, as fillagrin expression (a marker of terminal differentiation) was detected in the upper layer of the HaCat-IKKα skin equivalents of 12-day air-liquid culture, while at this time it was not expressed in the HaCaT-Control fibrin gels (Figure 2A). This result agrees with the presence of markers of terminal differentiation found in monolayer cultures of HaCaT-IKKα cells, where corneocytes were detected floating in the supernatant of keratinocytes overexpressing IKKα but not in that of HaCaT-Control cells [12]. Therefore our results show that increased levels of IKKα enhanced the stratification and differentiation of keratinocytes, although in a disorganized, not physiological manner.

### Invasive behavior and increased proliferation and clonogenic properties of the HaCaT-IKKα skin equivalents

In addition to the mentioned altered morphological features, we observed the appearance of invasive foci of keratinocytes growing into the underlying dermal compartment in the HaCaT-IKKα equivalents (Figure 2A, B arrow). This kind of growth was not observed in HaCaT-Control skin equivalents. We then analyzed the proliferation and apoptosis rates of the keratinocytes, finding that in those areas where the HaCaT-IKKα epidermis had lighter stratification defects, no differences in the number of BrdU positive cells were appreciated between keratinocytes of both genotypes (Figure 2C, 2D). However in those areas of the HaCaT-IKKα epidermal component where the phenotypic alterations were more evident (such as the invagination foci and the regions of higher disorganized stratification), increased BrdU signal was detected (Figure 2E–2G). This result agrees with the increased proliferation seen in keratinocytes of K5-IKKα mice [11], indicating a strong concordance between data obtained both *in vivo* in the skin of mice and in the human HaCaT-IKKα skin equivalents. The apoptosis rate was analyzed by cleaved Caspase 3 immunostaining and no differences were found between skin equivalents from the two genotypes of HaCaT cells (data not show).

**Figure 3:**
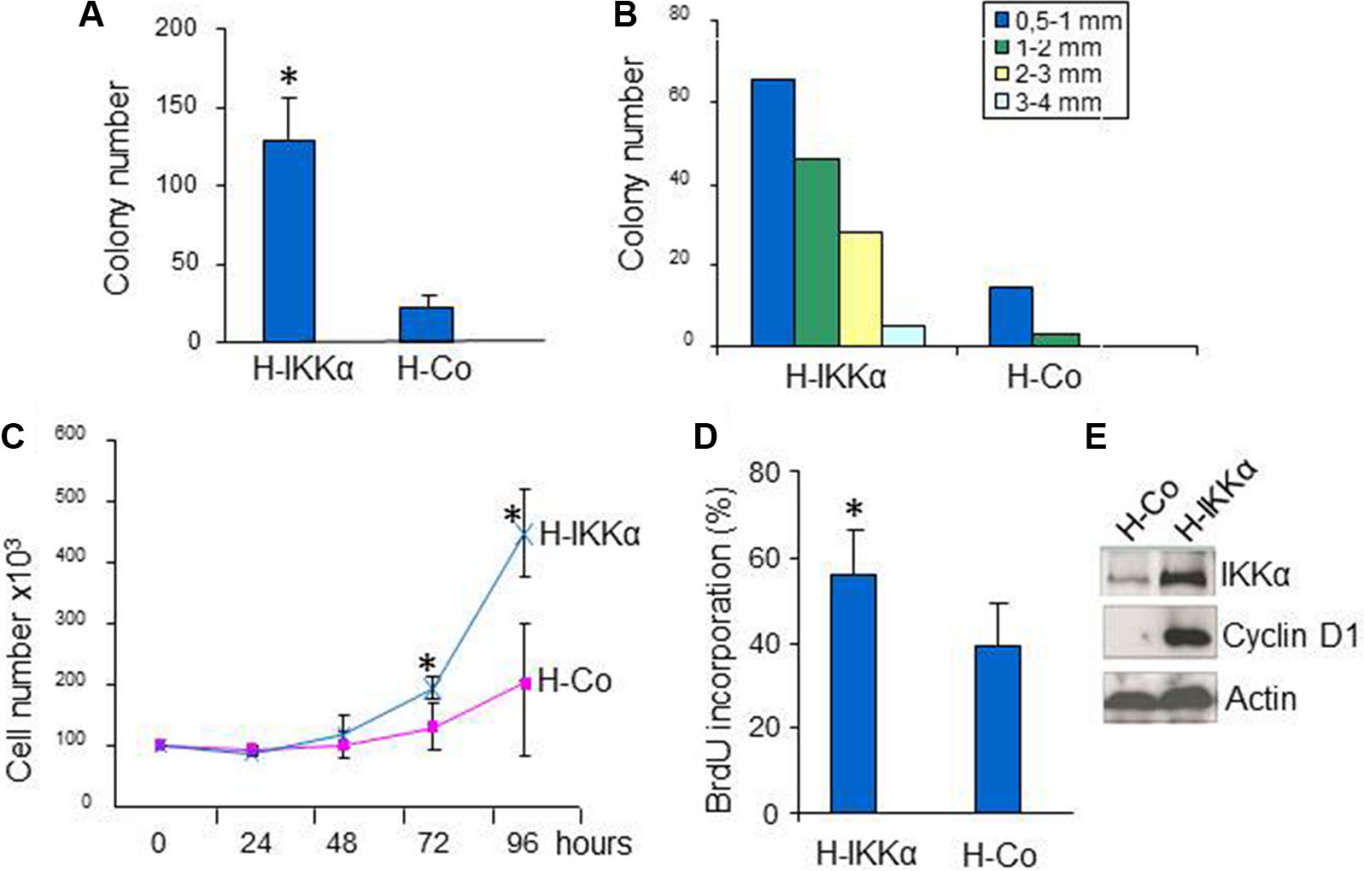
Increased growth in HaCaT-IKKα keratinocytes (**A**, **B**) Clonogenic assay. The mean of three different experiments is shown. (A) Total number of colonies grown 14-days after seeding HaCaT keratinocytes of both genotypes. (B) Representative example of size diameter distribution of colonies in a clonogenic assay. Colony size was measured using a magnifier. Colonies of diameter > 0.5 mm were counted and classified according to their diameter. (**C)** Growth curves of HaCaT-IKKα and HaCaT-Control keratinocytes. Three plates of cells were counted at the indicated times. Growth of HaCaT-IKKα cells was significantly higher at 72 and 96 h post seeding. (**D**) BrdU incorporation by HaCaT-Control and HaCaT-IKKα cells after 72 h of culture on coverslides. (**E**) Western blot showing the increased expression of CyclinD1 in HaCaT-IKKα cells. Student's *T* test was used for statistical analysis.*p < 0.05. H-IKKα = HaCaT-IKKα cells; H-Co = HaCaT-Control cells.

These characteristics of the HaCaT-IKKα skin equivalents, i.e., altered differentiation, increased proliferation and formation of invasion foci, along with the aberrant morphology of the epidermal compartment may be considered as premalignant signs. To further study the tendency towards malignant transformation of HaCaT-IKKα cells, we performed clonogenicity assays. Examination of the colony forming efficiency showed that HaCaT-IKKα cells produced higher number of colonies than HaCaT-Control cells (Figure 3A). In addition, clear differences were found in colony size: while all of the colonies originated by HaCaT-Control cells were equal or smaller than 2 mm of diameter, nearly 25% of the HaCaT-IKKα colonies had a diameter higher than 2 mm (Figure 3B). We also verified that HaCaT-IKKα cells grown in monolayer cultures exhibited increased proliferation, i.e. after 72 h of culture HaCaT-IKKα cells grew significantly faster than HaCaT-Control keratinocytes (Figure 3C); moreover the BrdU incorporation was also significantly higher in HaCaT- IKKα cells- indicating an enhanced proliferation- (Figure 3D). As a possible cause for this increase, we checked the expression of cyclin D1, one of the main cell cycle regulators that is positively regulated by IKKα [15], and found that it was induced in HaCaT-IKKα cells (Figure 3E). These results agree with our previous data showing increased expression of cyclin D1 in transgenic mice with exogenous expression of IKKα in the epidermis (K5-IKKα mice) [11].

### IKKα augments metalloprotease proteolytic activity of the HaCaT-IKKα skin equivalents and induces the expression of oncogenic proteins in HaCaT keratinocytes

**Figure 4:**
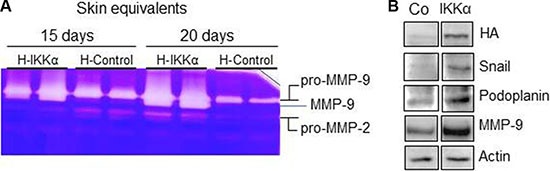
Increased metalloprotease proteolytic activity and increased expression of MMP-9, Snail and Podoplanin in the HaCaT-IKKα skin equivalents (**A**) MMP-2 and –MMP-9 activity was evaluated by gelatin zymography, using protein from culture supernatant of HaCaT-Control and HaCaT-IKKα skin equivalents at the indicated days of growth in air-liquid interface. White bands of proteolytic activity were revealed on a Coomassie-Blue stained gelatin gel. (**B**) Western blot showing the increased expression of Snail, Podoplanin and MMP-9 proteins in the HaCaT-IKKα skin equivalents grown for 12-days in air-liquid interface. Actin was used as a loading control.

By the time that the skin equivalents were collected, HaCaT-IKKα 3D cultures could be macroscopically distinguished from the HaCaT-Control skin equivalents by an evident reduction of the dermal compartment, i.e., a reduction in the thickness of the fibrin gel. This observation together with the above mentioned signs of invasiveness (Figure 2B) leaded us to study the proteolytic activity of MMP-2 and MMP-9, two matrix metalloproteases involved in the promotion of cancer cell invasion [16]. Increased proteolytic activity of MMP-9 was found in the HaCaT-IKKα skin equivalents by gelatin zymography, while MMP-2 activity did not change significantly (Figure 4A). This result was also confirmed by western blot analysis, using specific antibodies, which showed increased expression of MMP-9 metalloprotease (Figure 4B). Searching for a possible cause of this increase in MMP-9 levels we analyzed Snail, a positive regulator of MMP-9 expression that promotes cell invasion [17]. We found that Snail expression was induced in the HaCaT-IKKα skin equivalents, in comparison to the HaCaT-Control skin equivalents (Figure 4B). Another protein that accelerates cell motility and invasion of keratinocytes, is Podoplanin [18], a mucin-type transmembrane glycoprotein that is up-regulated in human squamous cell carcinomas of the skin [19]. The analysis of Podoplanin expression showed that it was highly induced in the HaCaT-IKKα skin equivalents (Figure 4B).

### Genetic profiling of HaCaT-Control and HaCaT-IKKα skin equivalents

To further identified genetic changes induced by IKKα overexpression in 3D cultures of human keratinocytes, we performed expression profiling on HaCaT-Control and HaCaT-IKKα skin equivalents (10 days). Total number of overexpressed and repressed genes was 122 and 88 respectively. Bioinformatic analysis based in gene function and ontology (Table 1), revealed a striking increase in the expression of genes related to keratinocyte differentiation, epidermis development and cornified envelope, such as *CNFN*; genes encoding small proline-rich proteins (*SPRR2C, SPRR3*); *CDSN; SCEL*; *KRTDAP*; *LCE3D; TGM1*, etc). There was also an upregulation of genes involved in establishing cell-cell junctions and desmosomes (*DSC2; DSG1; CDSN POF1B; CEACAM1; LMO7; OCLN…*). Specially remarkable is the upregulation in HaCaT-IKKα skin equivalents of genes involved in the development of various skin disorders, such as pruritus (*KLK5, KLK7*; *IL1β);* dermatitis (*BMP6*, TNFαIP6); eczema (*VNN3, IL1RL1, SPRR2B, SPRR3)*, psoriasis (*CDSN, KLK, LCN2, IL1β)*, palmoplantar keratoderma (*SLURP1*; *DSC2; DSG1*), and ichthyosis (*TGM1; ABCA12; ALOX12B, KLK5*). An upregulation of genes implicated in immune response (*DPP4, CD24; DEFB1; COLEC12; RFTN1; IL1β; RSAD2*) was also seen, which is interesting, as in addition to its well known function in epidermal homeostasis, IKKα has been previously related with immunity functions [20, 21]). Moreover, we observed increased expression of genes that are also overexpressed in different types of cancer (*SPINK7; BEX2, EDN1*, *CEACAM; PDPN; EREG; FBN1; CTHRC1; SLC6A14; IL1β* etc), among them we found upregulation of genes involved in the development of skin carcinomas (*LCN2; TNFRSF19 etc.*); head and neck squamous cell carcinomas (*CRTC1, CCNA1 (CYCLIN A1); EREG; NEFL; LCE3D*), and prostate cancer (*PDPN* (podoplanin), *LCN2*). In addition, although the number and function of downregulated genes seemed to be less relevant than those upregulated, however we have identified that some of them also appear downregulated in prostate cancer (*DST; VAV3; PLK3HH2; ITG6B; PPM1L*). The complete list of differentially expressed genes is shown in Table 2 and Supplementary Table S1. Therefore, these results obtained from the genetic profile analysis of HaCaT-IKKα skin equivalents agree with the histological and biochemical analysis presented above, as well as with our previous observations *in vitro* [12], altogether supporting the increased proliferation, the altered and enhanced terminal differentiation, and the invasive capacity observed in HaCaT-IKKα keratinocytes growing in 3D cultures. In addition microarrays analysis has also revealed a number of skin diseases and different types of cancer that may be related to the increased expression of IKKα.

**Table 1:** Bioinformatic analysis of microarrays based on gene function and ontology

UPREGULATED						
Category	Name	*p*-value	*q*-value FDR B&H	Hit Count in Query List	Hit Count in Genome	Hit in Query List
GO: Biological Process	keratinocyte differentiation	5.36E-13	1.41E-09	13	138	CRCT1,CDSN,EREG,CNFN,ABCA12,SPRR2B,SPRR2G,SPRR3,SCEL,TGM1,LCE3D,PRR9,ALOX15B
GO: Biological Process	epidermis development	3.72E-12	4.88E-09	17	340	CRCT1,CDSN,KLK7,EREG,CNFN,ABCA12,SPRR2B,SPRR2G,SPRR3,SCEL,TGM1,LCE3D,KRTDAP,PRR9,TNFRSF19,KLK5,ALOX15B
GO: Biological Process	T cell mediated immunity	9.44E-06	2.75E-03	6	90	DPP4,CEACAM1,RFTN1,RSAD2,P2RX7,IL1B
GO: Biological Process	immune response	2.89E-05	5.42E-03	22	1572	DPP4,KLK7,EREG,GBP1,LCN2,EDN1,CEACAM1,DEFB1,COLEC12,RFTN1,BMP6,RSAD2,P2RX7,CD24,RORA,IFI44L,IL1RL1,KLK5,IL1B,IL36G,MX1,MX2
GO: Biological Process	keratinocyte proliferation	1.32E-03	4.18E-02	3	40	EREG,SLURP1,TGM1
GO: Cellular Component	cornified envelope	7.49E-14	1.54E-11	10	48	CRCT1,CDSN,CNFN,SPRR2B,SPRR2G,SPRR3,SCEL,TGM1,LCE3D,PRR9
GO: Cellular Component	cell-cell junction	5.59E-08	5.76E-06	14	418	DPP4,CDSN,AOC1,DSC2,DSG1,SH3KBP1,TENM2,POF1B,CEACAM1,SLC5A1,TGM1,P2RX7,LMO7,OCLN
GO: Cellular Component	epidermal lamellar body	6.11E-07	4.19E-05	3	4	KLK7,ABCA12,KLK5
GO: Cellular Component	extracellular space	7.84E-06	4.04E-04	22	1449	COL6A3,KLK7,EREG,AOC1,SLURP1,CEACAM6,ADAMTS5,LCN2,EDN1,CEACAM1,DEFB1,BMP6,FBN1,PTPRG,A2ML1,CTHRC1,IL1RL1,KLK5,TNFAIP6,IL1B,IL36G,DKK2
GO: Cellular Component	desmosome	1.10E-05	4.52E-04	4	26	CDSN,DSC2,DSG1,POF1B
Human Phenotype	Palmoplantar keratoderma	1.35E-08	5.06E-06	7	60	DSC2,DSG1,ABCA12,SLURP1,TGM1,CYP4F22,ALOX12B
Human Phenotype	Congenital ichthyosiform erythroderma	2.74E-05	5.13E-03	3	12	ABCA12,TGM1,ALOX12B
Human Phenotype	Congenital nonbullous ichthyosiform erythroderma	4.50E-05	5.62E-03	3	14	TGM1,CYP4F22,ALOX12B
Pubmed	Association of psoriasis to PGLYRP and SPRR genes at PSORS4 locus on 1q shows heterogeneity between Finnish, Swedish and Irish families.	6.07E-06	1.63E-03	3	12	SPRR2B,SPRR2G,SPRR3
Gene Family	Interleukins and interleukin receptors	1.49E-03	1.19E-02	3	71	IL1RL1,IL1B,IL36G
Coexpression	Cluster c: genes identifying an intrinsic group in head and neck squamous cell carcinoma (HNSCC).	9.51E-27	5.81E-23	20	112	CRCT1,CDSN,KLK7,EREG,CALB1,CWH43,DSG1,ABCA12,SPRR2G,SLC6A15,NEFL,LCE3D,KRTDAP,CCNA1,PRR9,IL1RL1,KLK5,IL36G,ALOX12B,SLC6A14
Coexpression	Cluster e: genes identifying an intrinsic group in head and neck squamous cell carcinoma (HNSCC).	2.68E-13	1.64E-10	11	89	CEACAM5,SPRR2C,CEACAM7,SLURP1,SCEL,POF1B,TMEM45B,SPINK7,A2ML1,TMPRSS11D,ALOX15B
Coexpression	Human HeadandNeck_Toruner04_20genes	2.78E-07	3.54E-05	4	13	DSG1,SPRR3,CEACAM6,SCEL
Coexpression	Genes up-regulated in mice with skin specific double knockout of both RB1 and TP53 by Cre-lox.	3.39E-06	2.54E-04	13	601	EREG,TMEM45A,SH3KBP1,SLURP1,GBP1,CEACAM1,RSAD2,PTPRG,SULT2B1,KRTDAP,IL1RL1,TNFAIP6,IL36G
Coexpression	Genes down-regulated in epithelial prostate cancer cell lines over-expressing an oncogenic form of KRAS gene.	4.09E-06	2.63E-04	7	143	CWH43,SPRR3,SCEL,LCN2,EDN1,TGM1,SLC6A14
Coexpression	Genes up-regulated in epidermis after to UVB irradiation.	7.71E-06	4.62E-04	9	293	RHCG,DSC2,SPRR2B,TGM1,DHRS9,CD24,UPP1,CYB5R2,MX1
Coexpression	Genes up-regulated in hepatocellular carcinoma (HCC) from MYC and E2F1 double transgenic mice.	1.23E-04	3.87E-03	4	56	TMEM176B,IFI44,LCN2,DEFB1
Coexpression	Genes up-regulated upon knockdown of PTEN by RNAi.	2.26E-04	6.41E-03	6	189	SCEL,EDN1,DEFB1,RSAD2,CYB5R2,MX2
Coexpression	Genes up-regulated in RWPE-1 cells (prostate cancer) upon expression of constitutively active form of STAT3.	2.29E-04	6.46E-03	5	121	CALB1,PDPN,NEFL,NSG1,MX1
Coexpression	Up-regulated genes in the cancer gene signature, representing a gene signature of cellular transformation.	8.37E-04	1.58E-02	6	242	EREG,SLC2A3,SLC39A8,UPP1,IL1B,CYB5R2
Disease	Dermatologic disorders	1.43E-08	2.23E-05	14	371	DPP4,CDSN,KLK7,DSC2,DSG1,SPRR3,SLURP1,LCN2,POF1B,EDN1,TGM1,RORA,TNFAIP6,IL1B
Disease	Congenital Nonbullous Ichthyosiform Erythroderma	1.94E-06	1.01E-03	4	17	ABCA12,TGM1,CYP4F22,ALOX12B
Disease	Eczema	1.10E-05	3.43E-03	11	396	DPP4,COL6A3,KLK7,SPRR2B,SPRR3,VNN3,DEFB1,TGM1,IL1RL1,KLK5,IL1B
Disease	Squamous cell carcinoma	1.39E-05	3.62E-03	23	1608	EREG,CEACAM5,DSC2,DSG1,SPRR3,SH3KBP1,SYTL2,CEACAM7,ODC1,LCN2,EDN1,SLC2A3,PDPN,DEFB1,TGM1,SPINK7,C15orf48,CCNA1,CTHRC1,KLK5,IL1B,BEX2,ALOX12B
Disease	Pruritus	1.65E-05	3.68E-03	5	58	CDSN,KLK7,EDN1,KLK5,IL1B
Disease	Hyperkeratosis	2.48E-05	4.83E-03	5	63	CDSN,DSG1,ABCA12,TGM1,IL1B
Disease	Harlequin Fetus	3.43E-05	5.35E-03	3	12	ABCA12,TGM1,KLK5
Disease	Skin lesion	4.14E-05	5.87E-03	5	70	DSG1,EDN1,CEACAM1,DEFB1,IL1B
Disease	Psoriasis	1.68E-04	1.54E-02	12	629	CDSN,KLK7,VNN3,LCN2,EDN1,DEFB1,TGM1,LCE3D,PRR9,KLK5,IL1B,ALOX15B
Disease	Pancreatic carcinoma	2.23E-04	1.73E-02	21	1666	COL6A3,KLK7,CEACAM5,ERN1,CEACAM7,CEACAM6,ODC1,LCN2,CEACAM1,KRT23,PDPN,CCDC88A,PTPRG,P2RX7,NAV3,CD24,CTHRC1,OCLN,IL1B,FKBP5,DKK2
Disease	Dermatitis, Allergic Contact	2.72E-04	2.02E-02	5	104	SLC2A3,BMP6,UPP1,TNFAIP6,IL1B
Disease	Precancerous Conditions	5.32E-04	2.86E-02	9	423	COL6A3,CEACAM5,SYTL2,CEACAM7,ODC1,LCN2,CD24,IL1B,ALOX15B
Disease	Thyroid carcinoma	7.18E-04	3.49E-02	11	635	DPP4,CDSN,LCN2,EDN1,CEACAM1,PDPN,DEFB1,FBN1,P2RX7,NSG1,IL1B
Disease	Prostatic Hyperplasia	8.56E-04	3.60E-02	3	34	IFI44,EDN1,ALOX15B
Disease	Ichthyosis linearis circumflexa	1.19E-03	4.84E-02	3	38	KLK7,DSG1,KLK5
Disease	Skin Neoplasms	1.23E-03	4.84E-02	6	216	DPP4,DSC2,ODC1,LCN2,TNFRSF19,IL1B
Disease	Esophageal carcinoma	1.28E-03	4.84E-02	10	578	CEACAM5,DSC2,SPRR3,ODC1,LCN2,PDPN,BMP6,SPINK7,IL1B,ALOX15B
DOWNREGULATED						
Category	Name	*p*-value	*q*-value FDR B&H	Hit Count in Query List	Hit Count in Genome	Hit in Query List
GO: Molecular Function	receptor binding	1.43E-05	5.62E-03	18	1601	REEP1,NTRK2,CXCL14,CAT,EDIL3,ITGB6,LYPD1,DST,SEMA6D,LRP4,NCOA7,CCL2,PLAT,VAV3,MMP13,FYB,IL33,EPHA4
GO: Biological Process	response to wounding	3.52E-07	8.69E-04	16	967	SOX2,CFH,TNC,TFPI2,DST,CLU,CD36,SCGB1A1,PLA2G2A,CCL2,PLAT,VAV3,DUSP10,MMP12,IL33,EPHA4
GO: Biological Process	response to growth factor	5.82E-06	3.07E-03	12	671	SOX2,TNC,NTRK2,MECOM,CAT,RNF165,CLU,SCGB1A1,LRP4,CCL2,EEF2K,FGFR2
GO: Biological Process	MAPK cascade	1.39E-04	1.43E-02	12	928	SOX2,NTRK2,MECOM,CD36,MID1,PPM1L,PLA2G2A,CCL2,DUSP10,FGFR2,CTSH,EPHA4
GO: Biological Process	aging	9.75E-04	3.58E-02	6	308	NTRK2,CAT,RNF165,CLU,UCP2,CCL2
GO: Cellular Component	extracellular space	1.14E-07	3.10E-05	20	1449	CFH,PCSK5,SEPP1,TCN1,PLXDC1,TNC,OLFM4,CXCL14,CAT,CLU,CD36,SCGB1A1,APOL4,PLA2G2A,CCL2,PLAT,MMP13,CTSH,IL33,GABBR1
GO: Cellular Component	cytoplasmic, membrane-bounded vesicle	2.66E-07	3.10E-05	18	1237	PCSK5,SEPP1,HLA-DRA,OLFM4,CEMIP,DST,CLU,CD36,SCGB1A1,AP3M2,PLA2G2A,CCL2,PLAT,FGFR2,CTSH,PCDH7,IL33,GABBR1
GO: Cellular Component	secretory vesicle	3.21E-06	1.28E-04	11	535	PCSK5,SEPP1,OLFM4,CLU,CD36,SCGB1A1,PLA2G2A,PLAT,CTSH,PCDH7,GABBR1
Coexpression	Genes down-regulated in prostate cancer samples.	1.45E-11	8.28E-08	15	480	KRT15,KRT19,MECOM,ITGB6,FADS1,DST,SEMA6D,CLU,SCGB1A1,EEF2K,VAV3,FGFR2,PLEKHH2,PCDH7,IL33
Coexpression	Selected genes up-regulated in prostate tumors developed by transgenic mice overexpressing VAV3 in prostate epithelium.	3.21E-07	1.04E-04	6	89	CFH,TNC,ITGB6,CD36,PLAT,EPHA4
Coexpression	Genes with promoters occupied by SMAD2 or SMAD3 in HaCaT cells (keratinocyte) according to a ChIP-chip analysis.	3.95E-05	3.37E-03	11	823	KRT15,KRT19,TNC,TFPI2,EDIL3,LYPD1,FADS1,DST,CLU,CCL2,DUSP10
Coexpression	Genes down-regulated in metastatic tumors from the whole panel of patients with prostate cancer.	4.18E-05	3.37E-03	7	305	SOX2,THSD4,KRT15,CHRM3,PPM1L,FGFR2,PCDH7
Coexpression	Integrin, VEGF, Wnt and TGFbeta signaling pathway genes down-regulated in PC-3 cells (prostate cancer) after knockdown of PDEF by RNAi.	2.34E-04	1.05E-02	3	39	KRT19,PIK3R3,ITGB6
Disease	Malignant tumor of colon	1.07E-10	1.84E-07	27	1714	SOX2,CFH,SEPP1,KRT19,UBD,TNC,TFPI2,NTRK2,OLFM4,SLFN12,MECOM,CAT,EDIL3,CHRM3,ITGB6,CEMIP,LOC101930123,CLU,CD36,UCP2,PLA2G2A,CCL2,PLAT,EEF2K,FGFR2,MMP13,XPR1
Disease	Malignant neoplasm of pancreas	3.16E-06	6.83E-04	20	1619	SOX2,SEPP1,KRT19,TNC,TFPI2,NTRK2,OLFM4,CXCL14,BCKDHB,CLDN8,LOC101930123,CLU,CCL2,EEF2K,FGFR2,MMP12,IL33,GABBR1,EPHA4,XPR1
Disease	Atrophic condition of skin	7.78E-06	1.04E-03	6	118	CFH,KRT19,SLFN12,CLU,CCL2,FGFR2
Disease	Malignant neoplasm of skin	2.52E-04	7.27E-03	6	220	KRT19,CAT,PLAT,FGFR2,MMP12,IL33
Disease	Squamous cell carcinoma	4.35E-05	2.90E-03	18	1608	SOX2,KRT19,TNC,TFPI2,MECOM,CXCL14,CAT,ITGB6,DST,CLU,CD36,CCL2,PLAT,VAV3,FGFR2,MMP12,MMP13,IL33

**Table 2:** Genes differentially expressed in the HaCat-IKKα skin equivalents (for a complete list, see Supplementary inf, Table 1

Probe ID	Gene Symbol	Gene Title	FoldChange (log2)	*p*.value	Probe ID	Gene Symbol	Gene Title	FoldChange (log2)	*p*.value
223720_at	SPINK7	serine peptidase inhibitor, Kazal type 7 (putative)	2.37	0.0017	1569948_at	BC047651	Homo sapiens cDNA clone IMAGE:5275301.	−2.84	6.00E-04
220620_at	CRCT1	cysteine-rich C-terminal 1	2.24	2.00E-04	205413_at	MPPED2	metallophosphoesterase domain containing 2	−2.1	2.00E-04
205767_at	EREG	epiregulin	2.16	0.0047	214598_at	CLDN8	claudin 8	−2.03	0.0017
224329_s_at	CNFN	cornifelin	2.01	0.0015	212768_s_at	OLFM4	olfactomedin 4	−1.92	0.0015
224328_s_at	LCE3D	late cornified envelope 3D	1.94	0.0015	209821_at	IL33	interleukin 33	−1.91	0.0045
215465_at	ABCA12	ATP-binding cassette, sub-family A (ABC1), member 12	1.77	0.0024	201427_s_at	SEPP1	selenoprotein P, plasma, 1	−1.9	0.0044
204439_at	IFI44L	interferon-induced protein 44-like	1.69	0.0045	228038_at	SOX2	SRY (sex determining region Y)-box 2	−1.88	0.0054
219554_at	RHCG	Rh family, C glycoprotein	1.68	0.0024	203649_s_at	PLA2G2A	phospholipase A2, group IIA (platelets, synovial fluid)	−1.86	6.00E-04
211657_at	CEACAM6	carcinoembryonic antigen-related cell adhesion molecule 6 (non-specific cross reacting antigen)	1.67	0.0015	221796_at	NTRK2	neurotrophic tyrosine kinase, receptor, type 2	−1.84	0.0015
1564307_a_at	A2ML1	alpha-2-macroglobulin-like 1	1.66	0.0118	221795_at	NTRK2	neurotrophic tyrosine kinase, receptor, type 2	−1.81	0.0079
1554921_a_at	SCEL	sciellin	1.63	0.0015	222484_s_at	CXCL14	chemokine (C-X-C motif) ligand 14	−1.6	0.0185
205626_s_at	CALB1	calbindin 1, 28kDa	1.62	0.0051	204580_at	MMP12	matrix metallopeptidase 12 (macrophage elastase)	−1.57	0.0255
220664_at	SPRR2C	small proline-rich protein 2C (pseudogene)	1.61	0.0032	227266_s_at	FYB	FYN binding protein	−1.57	0.0024
236429_at	ZNF83	zinc finger protein 83	1.6	0.0015	218002_s_at	CXCL14	chemokine (C-X-C motif) ligand 14	−1.54	0.015
218990_s_at	SPRR3	small proline-rich protein 3	1.59	0.0034	1559633_a_at	CHRM3	cholinergic receptor, muscarinic 3	−1.42	0.0026
206193_s_at	CDSN	corneodesmosin	1.57	0.0143	211795_s_at	FYB	FYN binding protein	−1.31	0.0061
212531_at	LCN2	lipocalin 2	1.55	0.0015	225123_at	SESN3	sestrin 3	−1.31	0.0048
219795_at	SLC6A14	solute carrier family 6 (amino acid transporter), member 14	1.54	0.0127	228640_at	PCDH7	protocadherin 7	−1.31	0.0135
232056_at	SCEL	sciellin	1.53	0.0015	228766_at	CD36	CD36 molecule (thrombospondin receptor)	−1.29	0.0045
206884_s_at	SCEL	sciellin	1.52	0.0015	226420_at	MECOM	MDS1 and EVI1 complex locus	−1.27	0.0055
223484_at	C15orf48	chromosome 15 open reading frame 48	1.46	0.0071	204364_s_at	REEP1	receptor accessory protein 1	−1.25	0.0068
205625_s_at	CALB1	calbindin 1, 28kDa	1.41	0.0045	205890_s_at	GABBR1 /// UBD	gamma-aminobutyric acid (GABA) B receptor, 1 /// ubiquitin D	−1.21	0.0073
223082_at	SH3KBP1	SH3-domain kinase binding protein 1	1.4	0.0032	243546_at	–	–	−1.2	0.0079
1554168_a_at	SH3KBP1	SH3-domain kinase binding protein 1	1.39	0.0015	201860_s_at	PLAT	plasminogen activator, tissue	−1.19	0.0025
203234_at	UPP1	uridine phosphorylase 1	1.38	0.0091	1553705_a_at	CHRM3	cholinergic receptor, muscarinic 3	−1.18	0.0083
206176_at	BMP6	bone morphogenetic protein 6	1.37	0.0065	205725_at	SCGB1A1	secretoglobin, family 1A, member 1 (uteroglobin)	−1.16	0.0249
237732_at	PRR9	proline rich 9	1.37	0.0073	218806_s_at	VAV3	vav 3 guanine nucleotide exchange factor	−1.16	0.0034
203757_s_at	CEACAM6	carcinoembryonic antigen-related cell adhesion molecule 6 (non-specific cross reacting antigen)	1.36	0.0024	205959_at	MMP13	matrix metallopeptidase 13 (collagenase 3)	−1.15	0.0151
235438_at	–	–	1.36	0.0233	215692_s_at	MPPED2	metallophosphoesterase domain containing 2	−1.15	0.0055
206628_at	SLC5A1	solute carrier family 5 (sodium/glucose cotransporter), member 1	1.32	0.0024	201650_at	KRT19	keratin 19	−1.14	0.0032
201884_at	CEACAM5	carcinoembryonic antigen-related cell adhesion molecule 5	1.31	0.0036	242476_at	–	–	−1.14	0.0032
207381_at	ALOX12B	arachidonate 12-lipoxygenase, 12R type	1.31	0.0034	208228_s_at	FGFR2	fibroblast growth factor receptor 2	−1.13	0.0058
224009_x_at	DHRS9	dehydrogenase/reductase (SDR family) member 9	1.3	0.0028	221884_at	MECOM	MDS1 and EVI1 complex locus	−1.13	0.0125
235368_at	ADAMTS5	ADAM metallopeptidase with thrombospondin type 1 motif, 5	1.27	0.0213	208998_at	UCP2	uncoupling protein 2 (mitochondrial, proton carrier)	−1.12	0.0073
236119_s_at	SPRR2G	small proline-rich protein 2G	1.26	0.0082	225275_at	EDIL3	EGF-like repeats and discoidin I-like domains 3	−1.11	0.0295
203559_s_at	AOC1	amine oxidase, copper containing 1	1.25	0.0026	228108_at	PPM1L	protein phosphatase, Mg2+/Mn2+ dependent, 1L	−1.11	0.0182
205899_at	CCNA1	cyclin A1	1.25	0.0079	202295_s_at	CTSH	cathepsin H	−1.1	0.0496
223952_x_at	DHRS9	dehydrogenase/reductase (SDR family) member 9	1.25	0.0024	233289_at	–	–	−1.09	0.0384
1557321_a_at	CAPN14	calpain 14	1.23	0.0045	218807_at	VAV3	vav 3 guanine nucleotide exchange factor	−1.08	0.0446
238654_at	VSIG10L	V-set and immunoglobulin domain containing 10 like	1.23	0.0045	219885_at	SLFN12	schlafen family member 12	−1.07	0.03
216466_at	NAV3	neuron navigator 3	1.22	0.0079	227148_at	PLEKHH2	pleckstrin homology domain containing, family H (with MyTH4 domain) member 2	−1.05	0.0119
242773_at	SLC5A1	solute carrier family 5 (sodium/glucose cotransporter), member 1	1.22	0.0024	235683_at	SESN3	sestrin 3	−1.01	0.0255
208539_x_at	SPRR2B	small proline-rich protein 2B	1.2	0.0048	208083_s_at	ITGB6 /// LOC100505984	integrin, beta 6 /// uncharacterized LOC100505984	−1	0.0443
220723_s_at	CWH43	cell wall biogenesis 43 C-terminal homolog (S. cerevisiae)	1.2	0.0047	229553_at	PGM2L1	phosphoglucomutase 2-like 1	−1	0.0329
235272_at	SBSN	suprabasin	1.2	0.0068	218326_s_at	LGR4	leucine-rich repeat containing G protein-coupled receptor 4	−0.99	0.0223
221667_s_at	HSPB8	heat shock 22kDa protein 8	1.19	0.0036	228390_at	RAB30	RAB30, member RAS oncogene family	−0.99	0.0373
224367_at	BEX2	brain expressed X-linked 2	1.19	0.0346	242671_at	–	---	−0.99	0.0402
206199_at	CEACAM7	carcinoembryonic antigen-related cell adhesion molecule 7	1.14	0.0034	206488_s_at	CD36	CD36 molecule (thrombospondin receptor)	−0.98	0.0076
206714_at	ALOX15B	arachidonate 15-lipoxygenase, type B	1.13	0.0185	241684_at	–	–	−0.96	0.0475
218963_s_at	KRT23	keratin 23 (histone deacetylase inducible)	1.13	0.0036	242836_at	–	–	−0.96	0.0317
211478_s_at	DPP4	dipeptidyl-peptidase 4	1.12	0.0307	211922_s_at	CAT	catalase	−0.94	0.0188
202086_at	MX1	myxovirus (influenza virus) resistance 1, interferon-inducible protein p78 (mouse)	1.11	0.0061	233664_at	–	–	−0.94	0.0366
206953_s_at	LOC101927458 /// LPHN2	uncharacterized LOC101927458 /// latrophilin 2	1.11	0.0106	233882_s_at	SEMA6D	sema domain, transmembrane domain (TM), and cytoplasmic domain, (semaphorin) 6D	−0.94	0.0452
220724_at	CWH43	cell wall biogenesis 43 C-terminal homolog (S. cerevisiae)	1.1	0.0122	201645_at	TNC	tenascin C	−0.93	0.01

## DISCUSSION

Our model of HaCaT skin equivalents developed to study the function of IKKα in the epidermal homeostasis and in skin cancer has demonstrated to be a useful approach for these purposes. Our results show that IKKα promotes the rapid differentiation of keratinocytes in skin equivalents, confirming the fundamental role of IKKα in this process that has been previously reported [1, 3, 12]; moreover, our new model of HaCaT-IKKα skin equivalents has allowed us the sequential study of the epidermal stratification process, showing that keratinocytes overexpressing IKKα exhibit a pathological differentiation, displaying remarkable alterations in tissue stratification and keratinocyte orientation, suggesting that levels of expression of IKKα must be strictly regulated. Enhanced keratinocyte proliferation was also observed in the HaCaT-IKKα skin equivalents, probably due to an effort to counteract the fast differentiation rate of the suprabasal layers. This result is in agreement with the loss of tissue architecture that the epidermis of K5-IKKα transgenic mice displayed when it was exposed to proliferative stimulus (as cutaneous TPA treatment) [11]. Loss of epidermal tissue architecture is considered a premalignant signal and it is remarkable that additional signs of premalignancy have been detected in the HaCaT-IKKα skin equivalents, such as the expression of oncogenic proteins (Podoplanin, Snail, Cyclin D1); the increased expression and proteolytic activity due to MMP-9 increase; and the presence of foci of keratinocyte invasion. These features suggest the predisposition towards malignant transformation of keratinocytes overexpressing IKKα, and agree with our previous data showing that K5-IKKα transgenic mice presented an enhanced malignant potential for developing skin tumors [11]; they are also in accordance with our previous results showing the more aggressive phenotype of skin carcinomas arisen in immunodeficient mice injected with PDVC57 tumoral keratinocytes overexpressing IKKα [12].

Some of the proteins regulated by IKKα in the HaCaT-IKKα skin equivalents have been previously shown to be regulated by IKKα in different contexts, reinforcing the validity of our model. Among them it was reported that IKKα induced cyclin D1 in response to mitogens and DNA synthesis [15]. Here we show that the expression of Cyclin D1 is increased in HaCaT-IKKα keratinocytes that also display increased proliferation. Moreover, we detected augmented levels of Cyclin D1 in the skin of K5-IKKα mice that presented enhanced proliferation as well [11]. Induction of Snail by IKKα has been previously observed in pancreatic cancer cells where it was proposed that Snail was promoting EMT [22]; the induction of MMP-9 by IKKα has also been described in activated human leukocytes [23]. In addition, we have just reported that increased levels of IKKα only in the cytoplasm of both, human HaCaT keratinocytes and keratinocytes of the basal layer of transgenic mice, induce the expression of MMP-9 [24]. In all these cases IKKα has been proposed to enhance the malignancy of the different types of cells where it was expressed. Actually our recent results show the fast development of more aggressive skin tumors in chemical skin carcinogenesis performed in transgenic mice that overexpress IKKα only in the nucleus or in the cytoplasm of keratinocytes [24].

Data obtained in the analysis of the microarrays of HaCaT-Control and HaCat-IKKα skin equivalents showed upregulation of genes implicated in differentiation/keratinization, and epidermal barrier formation. These results are in agreement with our experimental studies presented above, indicating the enhanced capacity of terminal differentiation of HaCaT-IKKα keratinocytes (Figure 2). They also agree with our previous observations *in vitro* showing the accelerated and increased ability of terminal differentiation in monolayer cultures of keratinocytes overexpressing IKKα [12]. They are in agreement as well with data reported by other groups that describe the fundamental role of IKKα in epidermal IKKα morphogenesis and epidermal barrier formation [1–4]. In adition, our expression profiling studies in keratinocytes overexpressing IKKα show opposite results to those obtained in genetic profiling studies of IKKα-null keratinocytes, which showed the down-regulation of genes involved in keratinocyte terminal differentiation and epidermal barrier formation [1]. Other differences found between skin equivalents of both genotypes in our microarrays point to the upregulation in the HaCaT-IKKα equivalents of genes related to proliferation (such as Cyclin A1); proteolysis (i.e. proteinases of the kallikrein family); and genes implicated in invasiveness (such as podoplanin) (Figure 4 and Tables 1, 2, Supplementary Table S1). Thus, these results could support both the increased proliferation and invasive capacity found above in the HaCaT-IKKα 3D cultures (Figures 2, 3) and could explain the degradation of fibrin gels observed in the HaCaT-IKKα skin equivalents. We have also found changes in the genetic expression profile of HaCaT-IKKα skin equivalents of genes implicated in skin cancer development, but, in addition, changes in genes involved in the development and progression of other types of cancer, such as head and neck cancer and prostate cancer has been also established. Remarkably overexpression of IKKα in prostate cancer has been previously found to correlate with poor prognosis [25] and some studies have found a relationship between IKKα activation and head and neck cancer progression [26, 27].

It is interesting that many of the changes in gene expression found in HaCaT-IKKα skin equivalents are relative to genes involved in the development of different skin disorders (*CNFN, CDSN, TGM1, ALOX12B*, etc). In this regard, alterations in proteins of the differentiated and cornified layers of the skin (such as cornifelin, corneodesmosin, transglutaminase 1, alox12B, ABCA12 etc.), has been associated with various cutaneous pathologies in human, such as inflammatory diseases, i.e. psoriasis, eczema, dermatitis and ichthyosis [28–30]. This result agrees with the recent studies finding that the induction of IKKα has an important role in the pathogenesis of skin disorders that course with altered proliferation-differentiation equilibrium, such as psoriasis [8]. Moreover, microarrays analysis showed in HaCat-IKKα skin equivalents the upregulation of genes that are also upregulated in different types of ichthyosis (Table 1), and this is interesting, as several of the defects observed in IKKα mutant mice are similar to those manifested in human lamellar ichthyosis [3]. It is also noteworthy the differentially expressed genes found in HaCat- IKKα skin equivalents which are involved in the development of palmoplantar keratoderma, a heterogeneous group of disorders characterized by abnormal thickening of the palms and soles; being this phenotype consistent with the excesive differentiation displayed by the HaCat-IKKα skin equivalents.

Therefore, all these ocurrences reinforce the significance of our genetic profile studies and support the utility of our *in vitro* model of skin equivalents for studying the homeostasis and diseases of the skin. The relationship of IKKα overexpression with the development of different skin disorders and diverse types of cancer is an interesting issue to be investigated in future works.

Altogether, our results suggest that the increased expression of IKKα in human keratinocytes induces features of malignancy such as altered differentiation properties, increased proliferative and clonogenic properties, augmented ability for invasive growth, induction of the expression of oncogenic proteins and increased extracellular matrix proteolytic activity. All these characteristics make keratinocytes overexpressing IKKα to be at a higher risk to develop skin cancer than control keratinocytes.

## MATERIALS AND METHODS

### Plasmid constructs

The β-Actin-Control construct (containing the empty vector) and the β-Actin-HA-IKKα construct (containing the HA-tagged-murine IKKα cDNA, under the control of the β-Actin promoter), were previously described [12]. Both constructs confer resistance to G418.

### Cells and culture conditions

The HaCaT human keratinocyte cell line was grown in Dulbecco's modified Eagle's medium with Glutamax (Gibco-BRL, Gaithersburg, MD), supplemented with 10% FCS. HaCaT-Control cells (containing the β-Actin-Control construct) and HaCaT-IKKα cells (containing the β-Actin-HA-IKKα construct) were previously described [12]. Cells were permanently transfected using the calcium phosphate method. Resistant colonies were selected using G418 (0.45 mg/ml). Pools of HaCat-IKKα cells (derived from approximately 60 colonies) were employed in these experiments. For proliferation assays cells were incubated for 1h in presence of 10 mM BrdU.

### Colony forming assay

A total of 3 and 6 × 10^2^ cells were seeded per duplicate in DMEM-10% FCS in p100 plates. Medium was replaced every 4 days. Cells were fixed and stained with crystal violet. Experiments were performed three times.

### Cell proliferation assay

5 × 10^4^ cells/p60 were seeded in complete medium (DMEM-10% FCS). At 24, 48, 72 and 96 h cells were trypsinized and counted. Three experiments per triplicate were performed.

### Generation of skin equivalents

Primary human dermal fibroblasts were obtained from skin biopsies of health donors and were grown in DMEM containing 10% fetal calf serum, 2 mM glutamine, and epidermal growth factor (10 ng/ml). Donors provided informed consent for biopsy. Permission was obtained for specimens taken from organ donors. Skin equivalents were generated as described [31]. Briefly, 2.5 ml of fibrinogen solution (from cryoprecipitated pig blood) were added to 5 ml of keratinocyte growth medium containing 3 × 10^4^ dermal fibroblasts. Immediately later, 0.5 ml of 25 mM Cl_2_Ca, with 9 IU of bovine thrombin (Sigma-Aldrich Co., St. Louis) was added. The mixture was placed on polycarbonate inserts (4 μM porous) in a 6-well culture plate (Corning Costar Corp., Cambridge, MA) and allowed to solidify at 37°C for 45 min; after that, 10^6^ HaCaT cells were seeded on the fibrin matrix and grown to confluence. After reaching confluence the skin equivalent was raised to the air-liquid interface for variable time periods (up to 12 days) to generate a stratified epidermis. For proliferation assays skin equivalents were grown 1 h in presence of 10 mM BrdU.

### Histology and immunohistochemistry

Skin equivalents were fixed in 10% buffered formalin and embedded in paraffin. Sections were stained with H&E and histopathological evaluation of skin equivalents was performed by two specialists in pathological anatomy: MJFA, specialized in human pathology and AB, a veterinarian expert in animal pathology. Immunostaining was performed using antibodies against IKKα NB-100-56704 (Novus Biologicals, Cambridge UK); IKKα sc-7182 (Santa Cruz Biotechnology, Inc. Heidelberg, Germany); IKKα 556532, Plakoglobin (BD Bioscience, NJ, USA); Involucrin, Filaggrin (Covance, CA, USA); BrdU (Roche, Mannheim, Germany). Sections were incubated with a biotinylated secondary antibody, and then with streptavidin conjugated to horseradish peroxidase (DAKO A/S, Glostrp, Denmark). Antibody localization was determined using 3,3-diaminobenzidine (DAB) in H_2_O (Vector Laboratories; Burlingame, CA, USA).

Pressure cooker with DAKO target retrieval solution ph9.0 (DAKO) was employed for antibodies detection.

### Western blot analysis

Protein extracts were obtained from the epidermal compartment of skin equivalents. Total protein extracts (40 μg) were subjected to SDS/PAGE. The separated proteins were transferred to nitrocellulose membranes (Amersham, Arlington Heights, IL) and probed with antibodies against IKKα NB-100-56704 (Novus Biologicals); HA (Cell Signaling Technology, USA); Snail (Abcam, Cambridge, UK); Cyclin D1 (NeoMarkers, Fremont, CA, USA); Podoplanin, Actin (Santa Cruz Biotechnology); MMP-9 (Merck Millipore, Darmstadt, Germany). In all cases samples were subjected to luminography with the Supersignal West Pico Chemiluminescent Substrate (Pierce Biotechnology, Inc., Illinois, USA).

### Gelatin zymography

Gelatin zymography was performed as described [32]. 20 μg of protein extracts from supernanatants were subjected to SDS/PAGE with 0.1% gelatin (Sigma-Aldrich, MO, USA). Gel were stained with 0.25% Coomasie-Blue R-250 in methanol:acetic:water (5:1:5) and destained in 7.5% acetic acid.

### Microarrays of skin equivalents

Total RNA was isolated from the epidermal compartment of skin equivalents using TRIzol (Molecular Research Center Inc., Cincinnati, OH, USA) following manufacturer's instructions and DNA was eliminated using a DNAse column kit (Qiagen). HG-U133_Plus_2 arrays were used (Afymetrix) and annotations were updated to the last available version (June 2016). Data were normalized using RPA and processed using limma (Bioconductor). Genes with a foldchange of at least 1.5 and a *p value* of less than 0.05 were considered as regulated. Gene lists with up- or downregulated genes were submitted to ToppGene (as ranked lists) [33] or to JMP Enrichr (as fuzzy lists) [34] for enrichment analysis based on functional annotations.

## SUPPLEMENTARY MATERIALS TABLE




