# Correction: IKKα regulates the stratification and differentiation of the epidermis: implications for skin cancer development

**DOI:** 10.18632/oncotarget.20002

**Published:** 2017-08-07

**Authors:** Josefa P. Alameda, Manuel Navarro, Ángel Ramírez, Angustias Page, Cristian Suárez-Cabrera, Rodolfo Moreno-Maldonado, Jesús M. Paramio, María del Carmen Fariña, Marcela Del Río, María Jesús Fernández-Aceñero, Ana Bravo, María de los Llanos Casanova

**Present**: The grant support information is incomplete.

**Correct**: The complete grant support is as follows.

Original article: Oncotarget. 2016; 7:76779-76792. https://doi.org/10.18632/oncotarget.12527

**FUNDING**

This research was supported partially by funds from Fondo Europeo de Desarrollo Regional (FEDER) and by grants from the Ministry of Economy and Competitiveness of Spain (PI13/02580 and PI14/01403 from the Instituto de Salud Carlos III to M.L. Casanova and A. Ramírez respectively) and by grants grant 1.010.511 (Fundación Banco de Santander-Universidad Alfonso X el Sabio) Fernández-Aceñero; Comunidad Autónoma de Madrid grant S2010/BMD-2470 (Oncocycle Program) to JMP and CIEM13-4E-1944; to JMP AES grants ISCIII-RETIC RD06/0020/0029 and RD12/0036/0009 to JMP.

