# Supplementary material for: IKKα regulates the stratification and differentiation of the epidermis: implications for skin cancer development
Source: Oncotarget. 2016 Oct 8;7(47):76779–92. doi: 10.18632/oncotarget.12527 (PMC5363549; doi:10.18632/oncotarget.12527)
Supplement: Supplementary file 1 [file oncotarget-07-76779-s001.pdf]

IKKα regulates the stratification and differentiation of the epidermis: implications for skin cancer development

Supplementary Materials

Supplementary Table S1: Complete list of genes differentialy expressed in HaCaT-IKKα skin equivalents. See Supplementary_Table_S1
