# Supplementary material for: IKKα regulates the stratification and differentiation of the epidermis: implications for skin cancer development
Source: Oncotarget. 2016 Oct 8;7(47):76779–92. doi: 10.18632/oncotarget.12527 (PMC5363549; doi:10.18632/oncotarget.12527)
Supplement: Supplementary file 2 [file oncotarget-07-76779-s002.pdf]

Supp. Inf. Table 1. Complete list of genes differentialy expressed in HaCaT-IKKα skin equivalents

Probe ID	Gene Symbol	Gene Title	lChange (log2)	p.value
223720_at	SPINK7	serine peptidase inhibitor, Kazal type 7 (putative)	2,37	0,0017
220620_at	CRCT1	cysteine-rich C-terminal 1	2,24	2,00E-04
205767_at	EREG	epiregulin	2,16	0,0047
224329_s_at	CNFN	cornifelin	2,01	0,0015
224328_s_at	LCE3D	late cornified envelope 3D	1,94	0,0015
215465_at	ABCA12	ATP-binding cassette, sub-family A (ABC1), member 12	1,77	0,0024
204439_at	IFI44L	interferon-induced protein 44-like	1,69	0,0045
219554_at	RHCG	Rh family, C glycoprotein	1,68	0,0024
211657_at	CEACAM6	carcinoembryonic antigen-related cell adhesion molecule 6 (non-specific cross reacting antigen)	1,67	0,0015
1564307_a_at	A2ML1	alpha-2-macroglobulin-like 1	1,66	0,0118
1554921_a_at	SCEL	sciellin	1,63	0,0015
205626_s_at	CALB1	calbindin 1, 28kDa	1,62	0,0051
220664_at	SPRR2C	small proline-rich protein 2C (pseudogene)	1,61	0,0032
236429_at	ZNF83	zinc finger protein 83	1,6	0,0015
218990_s_at	SPRR3	small proline-rich protein 3	1,59	0,0034
206193_s_at	CDSN	corneodesmosin	1,57	0,0143
212531_at	LCN2	lipocalin 2	1,55	0,0015
219795_at	SLC6A14	solute carrier family 6 (amino acid transporter), member 14	1,54	0,0127
232056_at	SCEL	sciellin	1,53	0,0015
206884_s_at	SCEL	sciellin	1,52	0,0015
223484_at	C15orf48	chromosome 15 open reading frame 48	1,46	0,0071
205625_s_at	CALB1	calbindin 1, 28kDa	1,41	0,0045
223082_at	SH3KBP1	SH3-domain kinase binding protein 1	1,4	0,0032
1554168_a_at	SH3KBP1	SH3-domain kinase binding protein 1	1,39	0,0015
203234_at	UPP1	uridine phosphorylase 1	1,38	0,0091
206176_at	BMP6	bone morphogenetic protein 6	1,37	0,0065
237732_at	PRR9	proline rich 9	1,37	0,0073
203757_s_at	CEACAM6	carcinoembryonic antigen-related cell adhesion molecule 6 (non-specific cross reacting antigen)	1,36	0,0024
235438_at	---	---	1,36	0,0233
206628_at	SLC5A1	solute carrier family 5 (sodium/glucose cotransporter), member 1	1,32	0,0024
201884_at	CEACAM5	carcinoembryonic antigen-related cell adhesion molecule 5	1,31	0,0036
207381_at	ALOX12B	arachidonate 12-lipoxygenase, 12R type	1,31	0,0034
224009_x_at	DHRS9	dehydrogenase/reductase (SDR family) member 9	1,3	0,0028
235368_at	ADAMTS5	ADAM metallopeptidase with thrombospondin type 1 motif, 5	1,27	0,0213
236119_s_at	SPRR2G	small proline-rich protein 2G	1,26	0,0082
203559_s_at	AOC1	amine oxidase, copper containing 1	1,25	0,0026
205899_at	CCNA1	cyclin A1	1,25	0,0079

223952_x_at	DHRS9	dehydrogenase/reductase (SDR family) member 9	1,25	0,0024
1557321_a_at	CAPN14	calpain 14	1,23	0,0045
238654_at	VSIG10L	V-set and immunoglobulin domain containing 10 like	1,23	0,0045
216466_at	NAV3	neuron navigator 3	1,22	0,0079
242773_at	SLC5A1	solute carrier family 5 (sodium/glucose cotransporter), member 1	1,22	0,0024
208539_x_at	SPRR2B	small proline-rich protein 2B	1,2	0,0048
220723_s_at	CWH43	cell wall biogenesis 43 C-terminal homolog (S. cerevisiae)	1,2	0,0047
235272_at	SBSN	suprabasin	1,2	0,0068
221667_s_at	HSPB8	heat shock 22kDa protein 8	1,19	0,0036
224367_at	BEX2	brain expressed X-linked 2	1,19	0,0346
206199_at	CEACAM7	carcinoembryonic antigen-related cell adhesion molecule 7	1,14	0,0034
206714_at	ALOX15B	arachidonate 15-lipoxygenase, type B	1,13	0,0185
218963_s_at	KRT23	keratin 23 (histone deacetylase inducible)	1,13	0,0036
211478_s_at	DPP4	dipeptidyl-peptidase 4	1,12	0,0307
202086_at	MX1	myxovirus (influenza virus) resistance 1, interferon-inducible protein p78 (mouse)	1,11	0,0061
206953_s_at	LOC10192745	uncharacterized LOC101927458 /// latrophilin 2	1,11	0,0106
220724_at	CWH43	cell wall biogenesis 43 C-terminal homolog (S. cerevisiae)	1,1	0,0122
209267_s_at	SLC39A8	solute carrier family 39 (zinc transporter), member 8	1,06	0,0417
219935_at	ADAMTS5	ADAM metallopeptidase with thrombospondin type 1 motif, 5	1,06	0,0162
231577_s_at	GBP1	guanylate binding protein 1, interferon-inducible	1,06	0,0095
221805_at	NEFL	neurofilament, light polypeptide	1,04	0,0125
214536_at	SLURP1	secreted LY6/PLAUR domain containing 1	1,02	0,0068
244692_at	CYP4F22	cytochrome P450, family 4, subfamily F, polypeptide 22	1,02	0,0082
1555383_a_at	POF1B	premature ovarian failure, 1B	1,01	0,0091
207526_s_at	IL1RL1	interleukin 1 receptor-like 1	1	0,0201
214453_s_at	IFI44	interferon-induced protein 44	0,99	0,0136
232082_x_at	SPRR3	small proline-rich protein 3	0,99	0,0125
220230_s_at	CYB5R2	cytochrome b5 reductase 2	0,98	0,0429
232914_s_at	SYTL2	synaptotagmin-like 2	0,97	0,0109
239233_at	CCDC88A	coiled-coil domain containing 88A	0,97	0,0151
211883_x_at	CEACAM1	carcinoembryonic antigen-related cell adhesion molecule 1 (biliary glycoprotein)	0,96	0,0223
219908_at	DKK2	dickkopf WNT signaling pathway inhibitor 2	0,96	0,0219
233002_at	PPP4R4	protein phosphatase 4, regulatory subunit 4	0,96	0,0496
205759_s_at	SULT2B1	sulfotransferase family, cytosolic, 2B, member 1	0,95	0,0366
205778_at	KLK7	kallikrein-related peptidase 7	0,95	0,0125
236266_at	RORA	RAR-related orphan receptor A	0,95	0,0182
219799_s_at	DHRS9	dehydrogenase/reductase (SDR family) member 9	0,94	0,0214
206008_at	TGM1	transglutaminase 1	0,93	0,0162
222242_s_at	KLK5	kallikrein-related peptidase 5	0,93	0,0329

212646_at	RFTN1	raftlin, lipid raft linker 1	0,92	0,0151
227812_at	TNFRSF19	tumor necrosis factor receptor superfamily, member 19	0,92	0,0151
227180_at	ELOVL7	ELOVL fatty acid elongase 7	0,91	0,0294
231867_at	TENM2	teneurin transmembrane protein 2	0,91	0,0188
242625_at	RSAD2	radical S-adenosyl methionine domain containing 2	0,91	0,0161
1555416_a_at	ALOX15B	arachidonate 15-lipoxygenase, type B	0,9	0,0091
202674_s_at	LMO7	LIM domain 7	0,89	0,0146
230741_at	P2RX7	purinergic receptor P2X, ligand-gated ion channel, 7	0,87	0,0225
204750_s_at	DSC2	desmocollin 2	0,86	0,0158
224840_at	FKBP5	FK506 binding protein 5	0,86	0,0276
207602_at	TMPRSS11D	transmembrane protease, serine 11D	0,85	0,0211
214059_at	IFI44	interferon-induced protein 44	0,85	0,0402
235146_at	TMCC3	transmembrane and coiled-coil domain family 3	0,85	0,0225
206642_at	DSG1	desmoglein 1	0,84	0,0161
208650_s_at	CD24	CD24 molecule	0,84	0,049
206025_s_at	TNFAIP6	tumor necrosis factor, alpha-induced protein 6	0,83	0,0255
220528_at	VNN3	vanin 3	0,83	0,0188
225681_at	CTHRC1	collagen triple helix repeat containing 1	0,83	0,0364
209498_at	CEACAM1	carcinoembryonic antigen-related cell adhesion molecule 1 (biliary glycoprotein)	0,82	0,0441
210397_at	DEFB1	defensin, beta 1	0,82	0,0225
230835_at	KRTDAP	keratinocyte differentiation-associated protein	0,82	0,0188
206376_at	SLC6A15	solute carrier family 6 (neutral amino acid transporter), member 15	0,81	0,0151
221665_s_at	EPS8L1	EPS8-like 1	0,81	0,0287
1558846_at	PNLIPRP3	pancreatic lipase-related protein 3	0,8	0,0214
200790_at	ODC1	ornithine decarboxylase 1	0,8	0,0245
219410_at	TMEM45A	transmembrane protein 45A	0,8	0,0467
220532_s_at	TMEM176B	transmembrane protein 176B	0,8	0,0315
227126_at	PTPRG	protein tyrosine phosphatase, receptor type, G	0,8	0,048
229344_x_at	RIMKLB	ribosomal modification protein rimK-like family member B	0,76	0,0295
221019_s_at	COLEC12	collectin sub-family member 12	0,75	0,042
227492_at	OCLN	occludin	0,75	0,0462
227671_at	XIST	X inactive specific transcript (non-protein coding)	0,75	0,0329
230323_s_at	TMEM45B	transmembrane protein 45B	0,75	0,0294
202499_s_at	SLC2A3	solute carrier family 2 (facilitated glucose transporter), member 3	0,74	0,0463
221898_at	PDPN	podoplanin	0,74	0,0294
220322_at	IL36G	interleukin 36, gamma	0,73	0,0331
39402_at	IL1B	interleukin 1, beta	0,73	0,0329
201438_at	COL6A3	collagen, type VI, alpha 3	0,72	0,0423
204994_at	MX2	myxovirus (influenza virus) resistance 2 (mouse)	0,72	0,0366

212915_at	PDZRN3	PDZ domain containing ring finger 3	0,72	0,0441
221916_at	NEFL	neurofilament, light polypeptide	0,71	0,0487
223805_at	OSBPL6	oxysterol binding protein-like 6	0,71	0,0415
202766_s_at	FBN1	fibrillin 1	0,7	0,0454
209569_x_at	NSG1	neuron specific gene family member 1	0,69	0,0336
218779_x_at	EPS8L1	EPS8-like 1	0,69	0,035
235745_at	ERN1	endoplasmic reticulum to nucleus signaling 1	0,68	0,0423
222802_at	EDN1	endothelin 1	0,66	0,047
210653_s_at	BCKDHB	branched chain keto acid dehydrogenase E1, beta polypeptide	-0,63	0,0453
233386_at	---	---	-0,65	0,0429
1557545_s_at	RNF165	ring finger protein 165	-0,69	0,04
205285_s_at	FYB	FYN binding protein	-0,69	0,0377
203410_at	AP3M2	adaptor-related protein complex 3, mu 2 subunit	-0,7	0,032
237839_at	---	---	-0,7	0,0441
215501_s_at	DUSP10	dual specificity phosphatase 10	-0,71	0,0423
223366_at	ZNF704	zinc finger protein 704	-0,72	0,035
215986_at	---	---	-0,73	0,0366
225545_at	EEF2K /// LOC	eukaryotic elongation factor-2 kinase /// eukaryotic elongation factor 2 kinase-like	-0,73	0,0287
226035_at	USP31	ubiquitin specific peptidase 31	-0,74	0,0441
218885_s_at	GALNT12	polypeptide N-acetylgalactosaminyltransferase 12	-0,75	0,0339
225344_at	NCOA7	nuclear receptor coactivator 7	-0,75	0,0366
212942_s_at	CEMIP	cell migration inducing protein, hyaluronan binding	-0,76	0,0294
222835_at	THSD4	thrombospondin, type I, domain containing 4	-0,77	0,0225
227256_at	USP31	ubiquitin specific peptidase 31	-0,78	0,0295
1555600_s_at	APOL4	apolipoprotein L, 4	-0,79	0,0214
213800_at	CFH	complement factor H	-0,79	0,0188
226535_at	ITGB6 /// LOC	integrin, beta 6 /// uncharacterized LOC100505984	-0,8	0,0214
236934_at	---	---	-0,8	0,0295
238544_at	---	---	-0,81	0,0249
213652_at	PCSK5	proprotein convertase subtilisin/kexin type 5	-0,82	0,0487
222581_at	XPR1	xenotropic and polytropic retrovirus receptor 1	-0,82	0,0202
204455_at	DST	dystonin	-0,83	0,0225
242055_at	PSMG4	proteasome (prosome, macropain) assembly chaperone 4	-0,83	0,0342
205513_at	TCN1	transcobalamin I (vitamin B12 binding protein, R binder family)	-0,84	0,0127
208791_at	CLU	clusterin	-0,84	0,0151
209555_s_at	CD36	CD36 molecule (thrombospondin receptor)	-0,84	0,0267
221563_at	DUSP10	dual specificity phosphatase 10	-0,84	0,0135
227449_at	EPHA4	EPH receptor A4	-0,84	0,0244
1556253_s_at	---	---	-0,85	0,0199

212850_s_at	LRP4	low density lipoprotein receptor-related protein 4	-0,85	0,0301
215304_at	---	---	-0,85	0,0196
237035_at	RP11-319G9.3	---	-0,85	0,0329
208963_x_at	FADS1 /// MIF	fatty acid desaturase 1 /// microRNA 1908	-0,86	0,0168
219700_at	PLXDC1	plexin domain containing 1	-0,86	0,0441
208792_s_at	CLU	clusterin	-0,87	0,0151
209278_s_at	TFPI2	tissue factor pathway inhibitor 2	-0,87	0,0342
229901_at	ZNF488	zinc finger protein 488	-0,87	0,0454
202743_at	PIK3R3	phosphoinositide-3-kinase, regulatory subunit 3 (gamma)	-0,88	0,0469
208894_at	HLA-DRA	major histocompatibility complex, class II, DR alpha	-0,88	0,0151
203638_s_at	FGFR2	fibroblast growth factor receptor 2	-0,89	0,03
204734_at	KRT15	keratin 15	-0,89	0,0367
216598_s_at	CCL2	chemokine (C-C motif) ligand 2	-0,89	0,0294
234222_at	---	---	-0,89	0,0199
212909_at	LYPD1	LY6/PLAUR domain containing 1	-0,9	0,0151
203637_s_at	MID1	midline 1	-0,92	0,0151
201645_at	TNC	tenascin C	-0,93	0,01
214596_at	CHRM3	cholinergic receptor, muscarinic 3	-0,93	0,0423
218736_s_at	PALMD	palmdelphin	-0,93	0,0163
211922_s_at	CAT	catalase	-0,94	0,0188
233664_at	---	---	-0,94	0,0366
233882_s_at	SEMA6D	sema domain, transmembrane domain (TM), and cytoplasmic domain, (semaphorin) 6D	-0,94	0,0452
241684_at	---	---	-0,96	0,0475
242836_at	---	---	-0,96	0,0317
206488_s_at	CD36	CD36 molecule (thrombospondin receptor)	-0,98	0,0076
218326_s_at	LGR4	leucine-rich repeat containing G protein-coupled receptor 4	-0,99	0,0223
228390_at	RAB30	RAB30, member RAS oncogene family	-0,99	0,0373
242671_at	---	---	-0,99	0,0402
208083_s_at	ITGB6 /// LOC	integrin, beta 6 /// uncharacterized LOC100505984	-1	0,0443
229553_at	PGM2L1	phosphoglucomutase 2-like 1	-1	0,0329
235683_at	SESN3	sestrin 3	-1,01	0,0255
227148_at	PLEKHH2	pleckstrin homology domain containing, family H (with MyTH4 domain) member 2	-1,05	0,0119
219885_at	SLFN12	schlafen family member 12	-1,07	0,03
218807_at	VAV3	vav 3 guanine nucleotide exchange factor	-1,08	0,0446
233289_at	---	---	-1,09	0,0384
202295_s_at	CTSH	cathepsin H	-1,1	0,0496
225275_at	EDIL3	EGF-like repeats and discoidin I-like domains 3	-1,11	0,0295
228108_at	PPM1L	protein phosphatase, Mg2+/Mn2+ dependent, 1L	-1,11	0,0182
208998_at	UCP2	uncoupling protein 2 (mitochondrial, proton carrier)	-1,12	0,0073

208228_s_at	FGFR2	fibroblast growth factor receptor 2	-1,13	0,0058
221884_at	MECOM	MDS1 and EVI1 complex locus	-1,13	0,0125
201650_at	KRT19	keratin 19	-1,14	0,0032
242476_at	---	---	-1,14	0,0032
205959_at	MMP13	matrix metallopeptidase 13 (collagenase 3)	-1,15	0,0151
215692_s_at	MPPED2	metallophosphoesterase domain containing 2	-1,15	0,0055
205725_at	SCGB1A1	secretoglobin, family 1A, member 1 (uteroglobin)	-1,16	0,0249
218806_s_at	VAV3	vav 3 guanine nucleotide exchange factor	-1,16	0,0034
1553705_a_at	CHRM3	cholinergic receptor, muscarinic 3	-1,18	0,0083
201860_s_at	PLAT	plasminogen activator, tissue	-1,19	0,0025
243546_at	---	---	-1,2	0,0079
205890_s_at	GABBR1 /// U	gamma-aminobutyric acid (GABA) B receptor, 1 /// ubiquitin D	-1,21	0,0073
204364_s_at	REEP1	receptor accessory protein 1	-1,25	0,0068
226420_at	MECOM	MDS1 and EVI1 complex locus	-1,27	0,0055
228766_at	CD36	CD36 molecule (thrombospondin receptor)	-1,29	0,0045
211795_s_at	FYB	FYN binding protein	-1,31	0,0061
225123_at	SESN3	sestrin 3	-1,31	0,0048
228640_at	PCDH7	protocadherin 7	-1,31	0,0135
1559633_a_at	CHRM3	cholinergic receptor, muscarinic 3	-1,42	0,0026
218002_s_at	CXCL14	chemokine (C-X-C motif) ligand 14	-1,54	0,015
204580_at	MMP12	matrix metallopeptidase 12 (macrophage elastase)	-1,57	0,0255
227266_s_at	FYB	FYN binding protein	-1,57	0,0024
222484_s_at	CXCL14	chemokine (C-X-C motif) ligand 14	-1,6	0,0185
221795_at	NTRK2	neurotrophic tyrosine kinase, receptor, type 2	-1,81	0,0079
221796_at	NTRK2	neurotrophic tyrosine kinase, receptor, type 2	-1,84	0,0015
203649_s_at	PLA2G2A	phospholipase A2, group IIA (platelets, synovial fluid)	-1,86	6,00E-04
228038_at	SOX2	SRY (sex determining region Y)-box 2	-1,88	0,0054
201427_s_at	SEPP1	selenoprotein P, plasma, 1	-1,9	0,0044
209821_at	IL33	interleukin 33	-1,91	0,0045
212768_s_at	OLFM4	olfactomedin 4	-1,92	0,0015
214598_at	CLDN8	claudin 8	-2,03	0,0017
205413_at	MPPED2	metallophosphoesterase domain containing 2	-2,1	2,00E-04
1569948_at	BC047651	Homo sapiens cDNA clone IMAGE:5275301.	-2,84	6,00E-04

Fold change: Red: >2; orange: 1.5-2; light green: (-1.5)-(-2); green: < -2
